# Structural and oxidative investigation of a recombinant high-yielding fetal hemoglobin mutant

**DOI:** 10.3389/fmolb.2023.1133985

**Published:** 2023-03-16

**Authors:** Karin Kettisen, Maria Nyblom, Emanuel Smeds, Angela Fago, Leif Bülow

**Affiliations:** ^1^ Pure and Applied Biochemistry, Department of Chemistry, Lund University, Lund, Sweden; ^2^ Lund Protein Production Platform, Department of Biology, Lund University, Lund, Sweden; ^3^ Infection Medicine, Department of Clinical Sciences, Lund University, Lund, Sweden; ^4^ Anesthesiology and Intensive Care, Department of Clinical Sciences, Lund University, Lund, Sweden; ^5^ Zoophysiology, Department of Biology, Aarhus University, Aarhus, Denmark

**Keywords:** fetal hemoglobin, protein surface net charge, oxygen binding, redox reactions, DNA cleavage, X-ray crystallography

## Abstract

Human fetal hemoglobin (HbF) is an attractive starting protein for developing an effective agent for oxygen therapeutics applications. This requires that HbF can be produced in heterologous systems at high levels and in a homogeneous form. The introduction of negative charges on the surface of the α-chain in HbF can enhance the recombinant production yield of a functional protein in *Escherichia coli*. In this study, we characterized the structural, biophysical, and biological properties of an HbF mutant carrying four additional negative charges on each α-chain (rHbFα4). The 3D structure of the rHbFα4 mutant was solved with X-ray crystallography at 1.6 Å resolution. Apart from enabling a higher yield in recombinant protein production in *E. coli*, we observed that the normal DNA cleavage activity of the HbF was significantly lowered, with a four-time reduced rate constant for the rHbFα4 mutant. The oxygen-binding properties of the rHbFα4 mutant were identical to the wild-type protein. No significant difference between the wild-type and rHbFα4 was observed for the investigated oxidation rates (autoxidation and H_2_O_2_-mediated ferryl formation). However, the ferryl reduction reaction indicated some differences, which appear to be related to the reaction rates linked to the α-chain.

## 1 Introduction

To enable the design of a safe and functional oxygen therapeutic, a suitable oxygen-carrying component is essential. In humans, hemoglobin (Hb) is the oxygen-carrying protein responsible for transporting oxygen from the lungs to all tissues in the body. Due to the appropriate oxygen transport characteristics of human Hb, this protein has a central role in the development of a class of oxygen therapeutic products called hemoglobin-based oxygen carriers (HBOCs) (Alayash, 2014). No HBOC product to date has been approved for human use by key government agencies such as the European Medicines Agency (EMA) or the Food and Drug Administration (FDA). The persistent safety issues associated with HBOCs have been proposed to largely stem from toxic side effects of cell-free Hb, such as endothelial nitric oxide scavenging, oxidative side reactions, and heme-associated inflammatory responses (Buehler et al., 2010). These side effects are normally *in vivo* kept under control by inherent protective systems against hemolysis, including the plasma proteins haptoglobin, hemopexin, and α1-microglobulin (Alayash et al., 2013; Ascenzi et al., 2005). However, under more challenging conditions, for example, by applying significant amounts of extracellular Hb formulated as oxygen therapeutics, these systems can easily be overwhelmed and Hb-mediated toxicity will occur (Bozza and Jeney, 2020). To tackle these issues, significant efforts have been spent on engineering human Hb for HBOC use, both to clarify the origin of the toxic side reactions and to identify ways to control harmful activities (Varnado et al., 2013; Olson, 2020). Besides engineering the Hb molecule itself to achieve the required qualities, future development will also require efficient recombinant protein production systems capable of large-scale manufacture of such designed Hb molecules. Thus, an aspect to consider for efficient production of Hb in heterologous hosts is the protein design to enhance yield and stability during protein production.

Human hemoglobins are made up of four subunits, forming a heterotetramer of two α-type globins (α, ζ) and two β-type globins (β, γ, δ, ε) (Schechter, 2008). The most common variant, adult human hemoglobin (HbA, α_2_β_2_) has been used as a component to formulate many HBOCs. An alternative human protein is the fetal human hemoglobin (HbF), which carries the same α-subunits as HbA, but the α-subunits are paired with two γ-subunits instead of β-subunits. The γ- and β-subunit are largely similar but differ in their amino acid sequences at 39 or 40 residues (the γ-globin genes (HBG1 and HBG2) differ at one residue). The differences are manifested as a reduced sensitivity of the oxygen affinity to the allosteric effector 2,3-diphosphoglycerate (DPG) (Bauer et al., 1968; Bunn and Briehl, 1970), a higher alkali denaturation resistance (Perutz, 1974), and a lower tetramer-dimer dissociation constant (Yagami et al., 2002) compared to the adult protein. Recombinantly produced wild type (wt) HbA and wt HbF were compared side by side in terms of oxidative reactivity in a study by Simons et al. (2018), which concluded that both proteins have favorable qualities in diverse redox-related aspects, and from that perspective, neither Hb appeared more suitable than the other as an HBOC starting material. Still, the enhanced stability of HbF compared to HbA is an argument in favor of HbF as a more attractive candidate for large-scale production. Optimization of culture conditions can markedly improve HbF production and a lesser fraction of non-desirable Hb (consisting of e.g., the soluble β-type subunit homotetramers) were seen with HbF compared to HbA (Ratanasopa et al., 2016). We recently revisited a laboratory-scale protocol for shake flask HbF production and found that wt HbF yield in *Escherichia coli* flask cultures could be increased to ∼30 mg purified Hb/L (Kettisen and Bulow, 2021).

The most attractive host for recombinant Hb production has so far been *E. coli*, which comes with advantages such as ease of genetic manipulation and straightforward cultivation with cheap media components. However, this system also has drawbacks such as insufficient intrinsic heme supply for achieving high Hb expression, unsatisfactory methionine aminopeptidase activity, and endotoxin contamination. These concerns can for the most part be overcome, as heme supply in *E. coli* can be enhanced to improve the Hb yield (Weickert et al., 1999; Villarreal et al., 2008), methionine aminopeptidase can be co-expressed for efficient N-terminal Met removal (Shen et al., 1997), and endotoxin reduction can be carried out during downstream processing with commercial endotoxin-removal measures, or, alternatively, engineered *E. coli* strains with lower endotoxin levels may be considered (Mamat et al., 2015). As the limitations related to the heterologous host often can be managed, the area for improvement could be focused on the intrinsic properties of the protein. The α-subunit suffers from stability issues both in the red blood cells and during recombinant expression in *E. coli* (Hoffman et al., 1990). In the maturing red cell, the assembly of Hb is aided by the alpha-hemoglobin stabilizing protein (AHSP) acting as a chaperone (Weiss et al., 2005). This protein can also be co-expressed during Hb production in *E. coli* with favorable outcomes on the α-subunit yield (Vasseur-Godbillon et al., 2006), but protein engineering strategies to enhance the stability of the α-subunit during expression, as shown with the αG15A mutation (Graves et al., 2008), might, if explored more extensively, be similarly used to improve Hb production. The stability of the apoglobin has been shown to directly correlate with the expression yield of the monomeric model protein myoglobin (Samuel et al., 2015). Other strategies of increasing recombinant protein production have shown that adaptation of the protein surface to fit the cytosolic electrostatic environment of the host cell could significantly improve solubility (Mu et al., 2017). If also taking into account the correlation between increased surface net charge and myoglobin concentration in tissues of deep-diving mammals (Mirceta et al., 2013), the mutational plasticity of the Hb α-subunit, especially the exposed surface-located residues, is worthwhile to dissect further. As the subunits still must be able to readily combine with each other, as well as retain Hb functionality as an oxygen carrier, the choice of mutation sites for this strategy should focus on surface-exposed sites located far away from the subunit contact interfaces and key structures of the heme pocket. We have previously identified one HbF mutant that exhibited a significantly higher yield than recombinant wt HbF (wt rHbF) in *E. coli* cultures (Kettisen and Bulow, 2021). The present study aims to examine this particular HbF mutant in a more in-depth characterization study. The HbF mutant harbors substitutions of three positive lysine residues on the surface of the α-subunit into negatively charged glutamic acid, combined with an asparagine residue substituted for aspartate (Kettisen and Bulow, 2021). The mutation sites are spread out across the surface of the alpha chain at α11 (A9) Lys→Glu, α56 (E5) Lys→Glu [Hb Shaare Zedek (Abramov et al., 1980)], α78 (EF7) Asn→Asp [Hb J-Singa (Wong et al., 1984)], and α90 (FG2) Lys→Glu (Hb Sudbury). We analyze the consequences of introducing these negatively charged substitutions on the alpha chains of HbF in terms of oxygen binding, redox activity, heme loss, DNA cleavage, thermal stability and crystal structure. We have also included an initial study of plasma clearance in a simple mouse model.

## 2 Materials and methods

### 2.1 Production and purification of hemoglobin

Four mutations at surface-exposed locations on the α-subunit were previously selected for introducing negative charges on HbF (Kettisen and Bulow, 2021). The four mutations were as follows, α11 (A9) Lys→Glu, α56 Lys→Glu, α78 (EF7) Asn→Asp, and α90 (FG2) Lys→Glu, and this HbF variant will henceforth be called rHbFα4. The α- and γ-subunits were expressed in tandem using the HbF-pETDuet-1 vector as described previously (Ratanasopa et al., 2016).

The cells were grown in Terrific Broth supplemented with 0.1 mg/L carbenicillin for selective pressure, 0.1 mM IPTG for protein expression induction, and 1 mM δ-aminolevulinic acid to improve intracellular heme production. At the end of cultivation, the cultures were bubbled with carbon monoxide (CO) gas to stabilize the protein, and CO bubbling was repeated after each subsequent downstream processing step. The cultivation and purification procedures have been described in more detail elsewhere (Kettisen and Bulow, 2021). The purity of the Hb fractions was estimated with sodium dodecyl sulfate-polyacrylamide gel electrophoresis (SDS-PAGE) using densitometric analysis. Throughout the downstream procedures, the Hb concentration was estimated by scanning the spectra and calculating the target protein concentration based on the Hb-CO Soret peak at 419 nm. The purified samples were concentrated to 2 mM using Vivaspin^®^ 20, 30 kDa MWCO (Sartorius) and then snap-frozen in liquid nitrogen and stored at −80°C until further analysis. Before subsequent characterization experiments, heme-based Hb concentrations were confirmed using the pyridine-hemochromogen method (Antonini and Brunori, 1971). Isoelectric focusing was also performed in a Novex^®^ pH 3–10 IEF gel (Invitrogen) with IEF Standards (Bio-Rad), as well as under denaturing conditions in 8 M urea, using Immobiline DryStrip pH 3–10 (7 cm), run in an IPGphor Isoelectric Focusing System (Pharmacia) to verify the presence of two subunits.

### 2.2 Oxygen binding

Oxygen equilibrium curves were measured for the wt HbF and the HbFα4 mutant using a modified diffusion chamber (Weber, 1992) coupled to a gas mixing system (GMS500, Loligo Systems), as described previously (Jendroszek et al., 2018; Andersen et al., 2021). Briefly, pure oxygen (O_2_) and pure nitrogen gas (N_2_) were mixed to generate known pO_2_ at 37°C. The gas mixtures were used to equilibrate the atmosphere in the diffusion chamber containing the Hb sample. The samples were prepared in a concentration of 0.3 mM heme in 0.1 M HEPES buffer pH 7.4 supplemented with 0.1 M KCl and measured with or without the addition of 0.75 mM DPG (10x tetramer excess). The corresponding absorption change was measured at 415 nm (Hb-O_2_ Soret peak) and used to determine the fractional Hb-O_2_ saturation induced by changes in pO relative to the endpoints at full oxygenation and full deoxygenation during equilibration with pure O_2_ and N_2_, respectively. The oxygen affinity (P50) and cooperativity coefficient (n50) was determined from at least four saturation steps within 20%–80% Hb-O_2_ saturation range (Jendroszek et al., 2018).

### 2.3 Autoxidation

The spontaneous autoxidation reaction of ferrous Hb-O_2_ (Fe^2+^) to ferric Hb (Fe^3+^) was monitored for 60 h at 37°C. The samples were kept at 20 µM heme, in 100 mM phosphate buffer pH 7.4, supplemented with 4.6 U/mL superoxide dismutase, 414 U/mL catalase, and 1 mM EDTA. The spectrum was recorded in a Cary 60 UV–vis spectrophotometer (Agilent Technologies) every 15 min. In the end, the reaction was forced to completion by the addition of 1.5x excess potassium ferricyanide (Sigma-Aldrich) and after incubation with the oxidant, the endpoint spectrum was acquired. The collected spectral series were processed with component analysis in the 450–700 nm range (Singh et al., 2020), and the ferrous decay time course was fitted to a single exponential equation.

### 2.4 H_2_O_2_ oxidation

The rate of ferryl Hb (Fe^4+^) formation was examined by adding increasing concentrations of hydrogen peroxide to ferric Hb (Fe^3+^). The reaction was initiated by rapid mixing in a stop-flow setup using the RX-2000 Rapid Mixing Stopped-Flow Accessory (Applied Photophysics) coupled to a Cary 60 UV–vis spectrophotometer (Agilent Technologies) as described previously (Ratanasopa et al., 2015). Briefly, 1:1 mixing of 20 µM Hb (heme) in 40 mM phosphate buffer pH 7.2 with increasing concentrations of H_2_O_2_ in H_2_O, resulted in a final reaction concentration of 10 µM Hb (heme) with 100–500 µM H_2_O_2_ in 20 mM phosphate buffer. The ferric decay was monitored at 405 nm and fitted to a double exponential equation, and the fitted k_obs_ rates were plotted against the H_2_O_2_ concentration for determination of the second-order rate constant.

### 2.5 Ferryl reduction

The reverse reaction from the ferryl (Fe^4+^) to the ferric (Fe^3+^) state of Hb was studied by adding increasing concentrations of the reducing agent ascorbic acid at 25°C. The ferryl Hb sample was formed by incubating 5 µM ferric Hb in 40 mM phosphate buffer pH 7.2 with 20x H_2_O_2_ for 5 min and then removing the excess H_2_O_2_ by adding 12 U of catalase (Sigma-Aldrich) and waiting for 1 min. The reduction reaction was initiated by the addition of ascorbic acid at concentrations ranging from 0–500 µM. The spectral range between 450–700 nm was monitored during the time course for complete ferryl reduction to ferric Hb. The ferryl state of Hb is not stable and the protein will autoreduce to the ferric state spontaneously. The spontaneous reduction of the ferryl state was followed overnight and the obtained autoreduction rate was deducted from the time courses acquired with ascorbic acid. The time courses (545–630 nm) were fitted to double exponential equations, with the fast phase assigned to the α-subunit and the slow phase to the γ-subunit, as described before (Ratanasopa et al., 2015; Reeder et al., 2008; Cooper et al., 2019). The obtained rates were plotted against the ascorbic acid concentration and fitted with a rectangular hyperbola to find k_max_ and K_D_, plus a linear equation for the α-subunit k_obs_, while a single rectangular hyperbola was used to fit the γ-subunit k_obs_, as described by Simons et al. (2018).

### 2.6 Ferric reduction

The oxidized ferric (Fe^3+^) form can be reduced back to ferrous Hb (Fe^2+^) with reducing agents. The ferric form was prepared with potassium ferricyanide (Sigma-Aldrich) and buffer exchanged in PD-10 Desalting Column (GE Healthcare) to remove excess oxidant. 10 µM Hb (heme) in 40 mM phosphate buffer pH 7.2 was incubated with 10 mM ascorbate at 25°C. The spectral changes were monitored in the range 450–700 nm for 18 h. The kinetics were resolved with component analysis of the spectral range and the ferric decay was fitted to a single exponential equation to determine the ferric reduction rate.

### 2.7 Heme loss

Heme loss was monitored by incubating the heme scavenging myoglobin mutant H64Y/V67F (gMb) (Hargrove et al., 1994) with ferric samples of Hb. The reaction of 2.5 µM Hb (heme) with 30 µM gMb was monitored in the spectral range 450–700 nm for 12 h at 25°C in 20 mM phosphate buffer pH 7.2, supplemented with 150 mM sucrose, according to procedures described previously (Kettisen et al., 2018). The kinetic spectral series were resolved with component analysis and the time course was fitted to a double exponential equation to obtain the heme loss rates from the α- and γ-subunits.

### 2.8 DNA cleavage

The tendency of Hb to cleave DNA was assessed by examining the deterioration of purified pUC18 plasmid in presence of varying concentrations of Hb, according to previously described procedures, with some modifications (Chakane et al., 2017). The experiments were performed in 20 mM phosphate buffer pH 7.2 at 37°C, with a plasmid DNA (pDNA) concentration of 3 ng/µL and Hb concentrations ranging from 0 µM to 300 µM (heme). The incubation was performed in thin-walled PCR tubes in a total volume of 55 µL. Samples were withdrawn every hour for 6 hours, and a final sample at 12 h. 11 µL loading dye was added and mixed immediately before freezing the samples at −20°C. The samples were examined with gel electrophoresis in 1% agarose gels and densitometric analysis was used to quantitate the separated DNA bands. The nicking of the supercoiled pDNA resulted in the formation of the open circular pDNA band, and subsequently the linear pDNA, which in turn also deteriorated during the experiment at the highest Hb concentrations. The relative quantity of the supercoiled DNA band was plotted against time and fitted to a single exponential equation. The obtained rates were plotted against Hb concentration and a linear fitting to the data was used to determine the rate constant of the DNA decay.

### 2.9 Thermal denaturation

The thermal denaturation of Hb was monitored by differential scanning fluorimetry (DSF) in a Prometheus NT.48 instrument (NanoTemper Technologies). Ferrous Hb samples at 3 mg/mL (188 µM heme) in 100 mM phosphate buffer pH 7.4, either bound to O_2_-bound or CO, were analyzed in standard grade capillaries (Prometheus NT.48 Series nanoDSF Grade Standard Capillaries). The intrinsic fluorescence intensity ratios between 350 and 330 nm were measured over a temperature ramp from 20°C to 95°C. The data were processed in the PR.therm Control v.2.04 program and the onset and inflection temperatures were determined for each sample.

### 2.10 Size exclusion chromatography

The Hb samples were analyzed on a Superdex^®^ 75 10/300 GL column (GE Healthcare) in 50 mM sodium phosphate buffer pH 7.2, supplemented with 150 mM NaCl, at a flow rate of 0.5 mL/min. A standard curve containing conalbumin, carbonic anhydrase, ribonuclease, and aprotinin, was used to estimate protein size. Haptoglobin (Bio Products Laboratory) and ferric Hb were mixed in a 1:1 M ratio, incubated at 37°C for 30 min, and applied to the column in a total volume of 100 µL.

### 2.11 Animal study

The protocols used for the animal study were approved by the Institutional Animal Care and Use Committee at Malmö/Lund, Sweden (Kettisen et al., 2021). Briefly, endotoxin removal procedures were performed by passing the samples sequentially through two high-capacity *Proteus* NoEndo™ spin columns (Vivaproducts), according to the manufacturer’s instructions. Female Balbc mice (Janvier, Le Genest-Saint-Isle, France) were supplied with a top addition of Hb, injecting 5 mg of Hb (volume ≤0.2 mL). At five time-points (5 min, 2 h, 6 h, 8 h, and 24 h, n = 5) plasma and urine samples were collected and frozen and stored at −80°C before analysis. Physical data such as weight and body temperature were recorded. With four time-point groups per Hb variant plus non-injected control group (n = 5) for both wildtype and mutant HbF series, a total of 50 mice were used in this study. The samples were frozen and stored at −80°C before pharmacokinetic evaluation.

We used enzyme-linked immunosorbent assay (ELISA) to quantify the HbF levels in the samples. Rabbit anti-HbF affinity-purified polyclonal antibody (“Bonita”, Agrisera AB, Sweden), horseradish peroxidase-conjugated sheep anti-HbF polyclonal antibody (Bethyl Labs, TX, United States), and tetramethylbenzidine (TMB single solution, Invitrogen), were used to capture and detect the HbF protein in microtiter plates (NUNC, MaxiSorp, VWR). The absorbance was recorded at 650 nm using a SpectraMax^®^ M2 Microplate Reader (Molecular Devices LLC). A single exponential decay equation was fitted to the data with a least-square fitting using the Solver function in Excel (Microsoft) to determine the half-life in plasma. The mouse albumin levels in the urine samples were quantified with a commercial kit (Mouse Albumin SimpleStep ELISA Kit, Abcam, United Kingdom) to evaluate the effect on kidney filtration function.

### 2.12 Crystallization of the HbF mutant

Crystallization conditions were screened using a PACT premier™ crystallization screen (Molecular Dimensions) and a Mosquito (TTP Labtech, Melbourn, United Kingdom) crystallization robot for automated droplet formation (Newman et al., 2005). Crystals were grown at 20°C and hits were obtained in several conditions. After 10 days the crystals were harvested and cryoprotected with reservoir solution containing 20% (v/v) glycerol briefly before immersing in liquid nitrogen for cryostorage during transit for X-ray experiments. No measures were taken to prevent autoxidation of the heme and the final Hb crystals were present in the Met form. Diffraction data for rHbFα4 were collected at the MAX IV Laboratory (Lund, Sweden) at the BioMAX beamline (Ursby et al., 2020). The best data set was collected for a large crystal 50 µm × 150 µm × 600 µm) formed in 0.1 M Bis-Tris propane pH 6.5, 0.2 M sodium fluoride, 20% w/v PEG 3350 (Molecular Dimensions PACT premier screen MD1-29 condition F1) using a protein concentration of 10 mg/mL. The structure was solved by molecular replacement with PDB entries 1FDH (chain G for gamma chain) and 1BZ1 (chain A for alpha chain) as search models using PHASER of the PHENIX suite (Adams et al., 2010). Electron density and difference density maps were manually inspected, and the model was improved using Coot (Emsley et al., 2010) and several rounds of refinement using the Phenix.refine software (Adams et al., 2010). The calculation of R_free_ used 4.89% of the data.

### 2.13 Data and statistical analysis

All experimental reactions were performed in at least three repeats (n = 3) for each sample unless otherwise stated. The time courses were fitted with the Solver add-in in Microsoft Excel software using least square fitting. Statistical analysis was performed with independent t-tests (*p* < 0.05 was considered statistically significant) and the data are reported as mean ± SD.

## 3 Results

The recombinant HbF samples of wt rHbF and rHbFα4 were produced by expression in *E. coli* with shake flask cultures and successfully purified in two steps of liquid chromatography as described before (Ratanasopa et al., 2016; Kettisen et al., 2018; Kettisen and Bulow, 2021). The initial capture step was performed with a CaptoS resin (GE Healthcare) or a TREN resin (Bio-Works) for wt rHbF and rHbFα4, respectively. The final polishing step was performed on a Q HP resin for both protein variants. The yields of the final samples were similar to the values reported previously (Kettisen and Bulow, 2021), 30 mg/L and 69 mg/L for wt rHbF and mutant rHbFα4, respectively. Isoelectric focusing were used to determine the effects of the introduced surface charges. The obtained pI values for the mutant was 5.8 for compared to 7.1 for the wt HbF. The isoelectric focusing study clearly verifies that the mutations contribute to lowering the pI of the HbF mutant.

**FIGURE 1:**
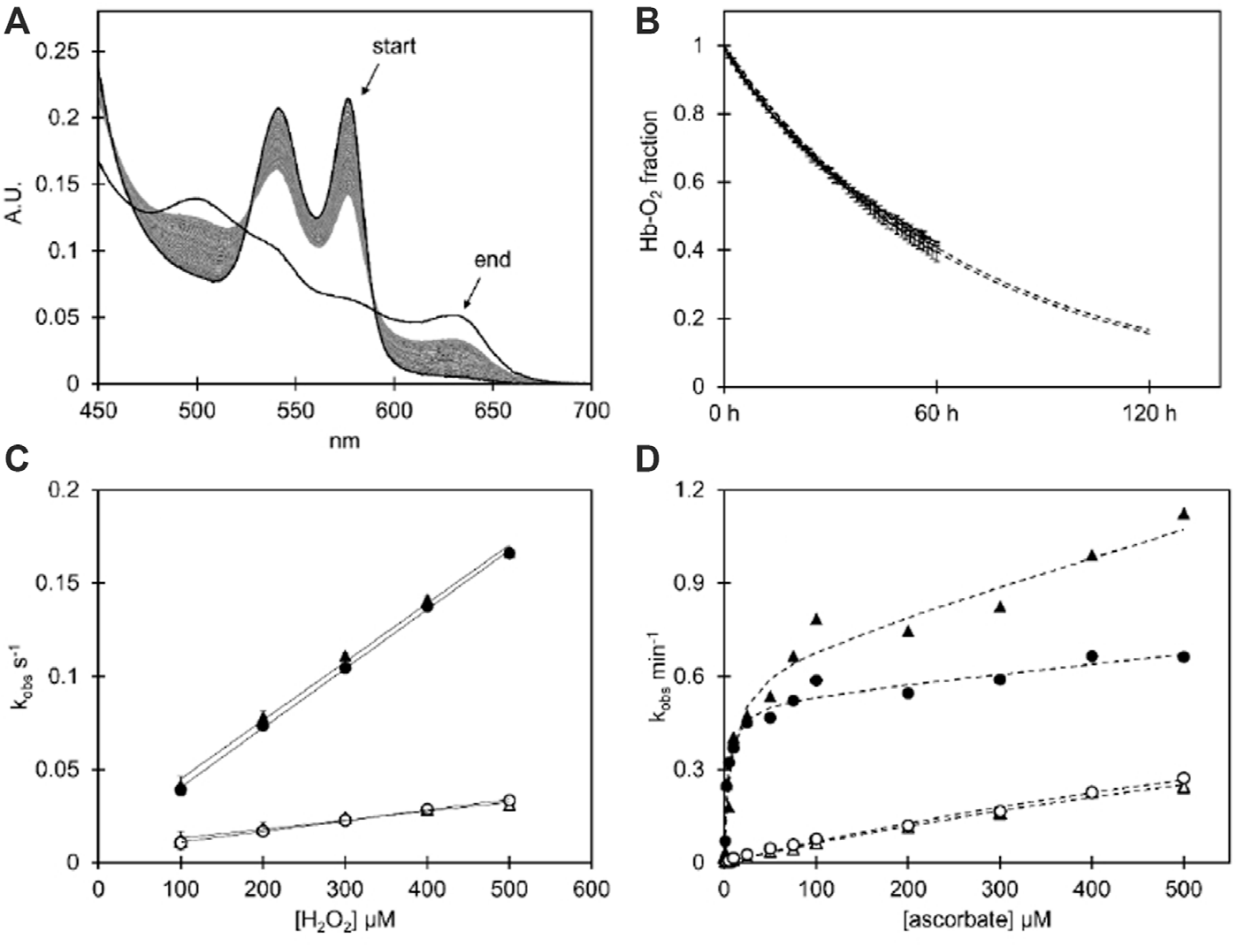
**(A)** Spectral series of the spontaneous autoxidation reaction of the HbFα4 mutant during 60 h at 37°C in 100 mM phosphate buffer pH 7.4 supplemented with 4.6 U/mL superoxide dismutase, 414 U/mL catalase, and 1 mM EDTA using Hb at 20 µM heme. The end spectrum shows the potassium ferricyanide-induced complete ferric state of the same sample. **(B)** The autoxidation decay of ferrous O_2_-bound Hb as resolved by component analysis for wt HbF and the HbFα4 mutant. The data is shown with error bars representing the standard deviation (n = 3), and the fitted single exponential decay equation is depicted as dashed lines, which extend beyond 60 h to represent the extrapolated continued reaction. **(C)** The graph shows the k_obs_ plotted against the H_2_O_2_ concentration of the H_2_O_2_-induced ferryl formation experiment (Fe^3+^ → Fe^4+^), with a linear fitting of the second-order rate constant µM^−-^s^-1^. **(D)** The plot shows the k_obs_ plotted against ascorbate concentration during the induced reduction from the ferryl to the ferric state of Hb (Fe^4+^ → Fe^3+^). The data is fitted with a rectangular hyperbola plus a linear equation for the fast phase (α-subunit), and a single hyperbola for the slow phase (γ-subunit). For graphs **(C, D)**, the fast phase k_obs_ are shown with filled symbols and the slow k_obs_ have open symbols. Circles represent wt HbF and triangles represent HbFα4 mutant. For experimental details cf Material and Methods.

The redox behavior of the two HbF variants was monitored during spontaneous reactions and by examining the consequences of oxidant/reductant agent additions. A summary of the experimental results obtained can be found in Table 1. Overall, the introduction of negative charges appeared to have no or only limited effects on the redox properties. The autoxidation rates did not differ between the rHbFα4 mutant and the wt rHbF (Figures 1A, B). The same was concluded for the ferryl formation rates with H_2_O_2_, (Figure 1C), as the calculated minor difference in rates was not regarded as significant. The reduction rates from the ferryl and ferric state by ascorbic acid, however, did show some more prominent differences (Figure 1D). The autoreduction rate of the rHbFα4 mutant from the ferryl to the ferric state was doubled in the fast phase, and increased by 60% in the slow phase, compared to wt rHbF (Table 1). This indicated that the mutant more quickly reverted to the ferric state than wt rHbF when no reducing agent was present. However, when ascorbate was added, no significant differences were found in the slow phase of the reaction between the two HbF variants. The slow kinetic phase has previously been assigned to be governed by the γ-subunit (Reeder et al., 2008; Cooper et al., 2019). In contrast, the faster phase governed by the α-subunit showed that the mutant was less efficiently converted to the ferric state compared to wt rHbF, as seen by the k_max_/K_D_ value, which was only 60% of wt rHbF (Table 1). However, the low-affinity phase (direct heme reduction of the α-subunit) showed a significantly faster rate for the mutant HbF. The ferric reduction experiment (Fe^3+^ to Fe^2+^) with ascorbate also showed that the mutant converted back to the ferrous state more slowly than the wt rHbF, further suggesting that the mutant utilized the reducing agent less efficiently. In summary, the oxidation rates towards the oxidized iron states ferric (Fe^3+^) and ferryl (Fe^4+^) did not differ between wt rHbF and the mutant. The reduction of the heme iron from both ferryl to ferric, and ferric to ferrous, indicated that the mutant less efficiently utilized the reducing power of ascorbate compared to wt rHbF. The observed differences appeared to be related to the kinetic phases directed by the α-subunit. Although, the mutant did show a faster autoreduction rate from the ferryl to the ferric state in absence of ascorbic acid, as well as a faster slow phase (direct heme reduction) rate in the α-subunit in presence of ascorbate.

**TABLE 1:** Summary of the redox reaction rates, heme loss, and DNA cleavage of the wt rHbF and the rHbFα4 mutant.

			wt rHbF	rHbFα4
Autoxidation rate (*h* ^*-1*^)			0.0142 ± 0.0002	0.0154 ± 0.0009
Ferryl formation by H_2_O_2_ (*µM* ^*-1*^ *s* ^*-1*^)		k_α_	0.6 ± 0.01 10^−4^	0.5 ± 0.01 10^−4^
		k_γ_	3.2 ± 0.01 10^−4^	3.1 ± 0.01 10^−4^
Ferryl reduction				
α-subunit	(*min* ^*-1*^)	k_max_	0.52 ± 0.02	0.63 ± 0.08
	(*mM*)	K_D_	3.8 ± 0.2 10^−3^	7.9 ± 3.1 10^−3^
	(*mM* ^*-1*^ *min* ^*-1*^)	k_max_/K_D_	138 ± 6	80 ± 17
	(*min* ^*-1*^)	k_linear_	3.1 ± 0.2 10^−4^	9.0 ± 2.0 10^−4^
	(*min* ^*-1*^)	k_autored_	0.07 ± 0.004	0.14 ± 0.005
γ-subunit	(*min* ^*-1*^)	k_max_	0.98 ± 0.11	0.99 ± 0.30
	(*µM*)	K_D_	1.3 ± 0.2	1.5 ± 0.6
	(*mM* ^*-1*^ *min* ^*-1*^)	k_max_/K_D_	0.73 ± 0.04	0.67 ± 0.08
	(*min* ^*-1*^)	k_autored_	0.010 ± 0.0015	0.016 ± 0.0022
Ferric reduction (*h* ^*-1*^)			0.26 ± 0.02	0.21 ± 0.01
Heme loss rate (*min* ^*-1*^)		k_α_	5.3 ± 0.06 10^−3^	6.1 ± 0.04 10^−3^
		k_γ_	57 ± 2 10^−3^	60 ± 7 10^−3^
DNA cleavage rate constant (*µM* ^*-1*^ *h* ^*-1*^)			15.7 ± 0.5 10^−3^	3.6 ± 0.02 10^−3^

The heme release from the ferric state of the HbF variants was monitored with the heme scavenging myoglobin mutant H64Y/V67F. The fast phase governed by the γ-subunit (k_γ_) was found not to differ between the samples, while the slow phase (k_α_) did show a minor significant difference, with a slightly faster heme release from the mutant compared to wt rHbF (Table 1).

**FIGURE 2:**
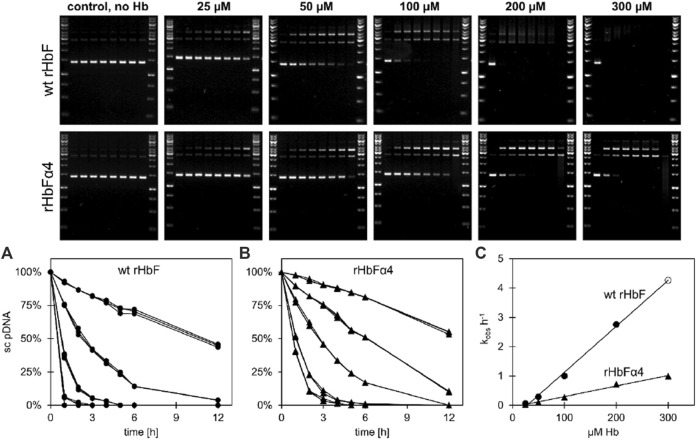
Plasmid DNA cleavage of wt HbF and the HbFα4 mutant was monitored for 12 h at 37°C in 20 mM phosphate buffer pH 7.2. Without the addition of Hb, no cleavage of the supercoiled (sc) pDNA was observed. In contrast, when Hb was included, the sc form deteriorated and open circular and linear pDNA bands emerged. By increasing the Hb concentrations from 0 µM to 25, 100, 200 and 300 µM (heme) the process was accelerated. The decay of sc pDNA over time was plotted and fitted to a single exponential equation and the data for wt HbF and HbFα4 mutant can be seen in graphs **(A**) and **(B)**, respectively. Circles represent wt HbF and triangles represent HbFα4 mutant. The obtained rates were then plotted against the Hb concentration and the linear relationship was used to determine the decay constants of pDNA cleavage [graph **(C)**]. The data point for wt HbF at 300 µM (open circle) in graph **(C)** is a product of the linear fitting to the other wt HbF data points, due to the lack of detection of the sc DNA band after 1 h of incubation.

Hb is prone to participate in oxidative side reactions causing modifications of other biomolecules like lipids, proteins, and nucleic acids. Hydrolytic cleavage of DNA caused by oxygen radicals formed by Hb is thus often observed (Chakane et al., 2017). The DNA cleavage activity can be analyzed by monitoring the decay of the supercoiled plasmid DNA in presence of increasing concentrations of Hb (Figure 2). Prominent differences were observed between the two Hb variants. When comparing the two HbF proteins, the rate constant for the mutant HbF was only 23% of the wt rHbF, indicating a much slower DNA degradation for the modified Hb (Figure 2C). This verified that the mutant HbF protein significantly reduced the degradation activity on the DNA backbone in comparison to wt rHbF. At the highest Hb concentration used in this study, 300 µM, the supercoiled DNA band completely disappeared after only 1 h when incubated with the wt rHbF. In contrast, at the same concentration of the mutant, 40% of the supercoiled DNA band remained after 1 h, and the signal could be detected up to 4 h before disappearing.

Thermal denaturation of the HbF proteins was measured using DSF by monitoring the ratio 350/330 nm. This technique measures the changes in fluorescent side-chain exposure of tryptophan and tyrosine during unfolding events. Three temperature ramps were used to assess the structural movements. We found that at slower temperature ramps, unfolding events were difficult to resolve for the wt rHbF due to 350/330 nm ratio measurements resulting in complicated first derivative plots (Supplementary Figure S1). This could be remedied with faster temperature ramps. Overall, we found that the amplitude of the 350/330 nm ratio change was markedly lower for the wt rHbF in comparison to the rHbFα4 samples. Nevertheless, the resolved thermal denaturation events showed no significant differences between the wt rHbF and the rHbFα4 mutant (Table 2). As for the results regarding the different ligand-bound samples, the CO-bound samples had two distinct transition temperatures, while O_2_-bound samples only displayed a single well-defined transition temperature. The first transition of the CO-bound samples overlapped with the peak of the O_2_-bound samples, and the corresponding scattering curves showed that this transition temperature was accompanied by a significant increase in scattering signal in the O_2_-bound samples, probably signifying a tandem collapse of the protein structure and globin precipitation. This increase in scattering did not appear in the CO-bound samples at the first transition temperature but appeared later at the second transition temperature. This implied a delay of the collapse when CO was bound to the protein in comparison to when the Hb was carrying O_2_. In the end, when comparing the calculated temperatures for both proteins no significant differences in neither onset nor transition temperatures were seen between the wild type and the mutant HbF samples.

**TABLE 2:** Thermal denaturation data of wt and mutant HbF as determined by nanoDSF in a Prometheus NT.48 instrument (NanoTemper Technologies).

	wt HbF		HbFα4	
	O_2_	CO	O_2_	CO
Onset	55.5°C ± 1.5°C	55.4°C ± 2.4°C	53.7°C ± 0.3°C	54.6°C ± 0.7°C
1^st^ peak	62.9°C ± 1.3°C	65.1°C ± 1.3°C	62.6°C ± 0.8°C	62.2°C ± 0.5°C
2^nd^ peak	N/A	76.7°C ± 0.9°C	N/A	75.8°C ± 0.7°C

Haptoglobin (Hp) is a plasma protein that captures cell-free Hb with high affinity. It has an important physiological role and by forming a Hp-HbF complex it inhibits toxic oxidative reactions linked to Hb (Alayash et al., 2013). To verify that the negative charges of the mutant HbF did not disrupt this binding reaction, the Hb samples were applied to a size exclusion column, with and without prior incubation with Hp (Supplementary Figure S2). The wt rHbF and the rHbFα4 mutant eluted at the same volumes, both when analyzed in their free forms as well as when complexed to Hp. The incubation with Hp demonstrated that there were no apparent changes to the dimer interface that participates in the binding of Hp for the rHbFα4 mutant.

The toxicity and plasma half-life of the HbF mutant was assessed in a top-load mouse model experiment. All test animals appeared healthy after injection of either wildtype or mutant HbF, and no significant differences in weight or body temperature were found compared to the untreated controls. The plasma samples were examined with sandwich ELISA to assess the Hb concentration in plasma and urine and showed decreasing concentrations throughout the experiment (Supplementary Figure S4). The proteins were relatively quickly removed from circulation and did not remain in the plasma at detectable levels after 24 h. Both proteins showed an identical behavior. To further characterize plasma clearances, Hb levels were also analyzed in urine samples. The Hb concentrations reached a peak after 2 h and then slowly dropped to background levels after 24 h. The calculated half-life was 36 min, indicating a fast clearance of the Hb protein from the plasma. The albumin analysis in the urine samples showed that albumin levels were elevated at 2, 6, and 8 h, while at 24 h the albumin was back to the level of the untreated control. The increased albumin level in urine indicated that the overall glomerular filtration was affected by the Hb proteins during the experiment. However, the albumin level decreased back to non-injected control levels at 24 h, showing that the effect was transient.

**FIGURE 3:**
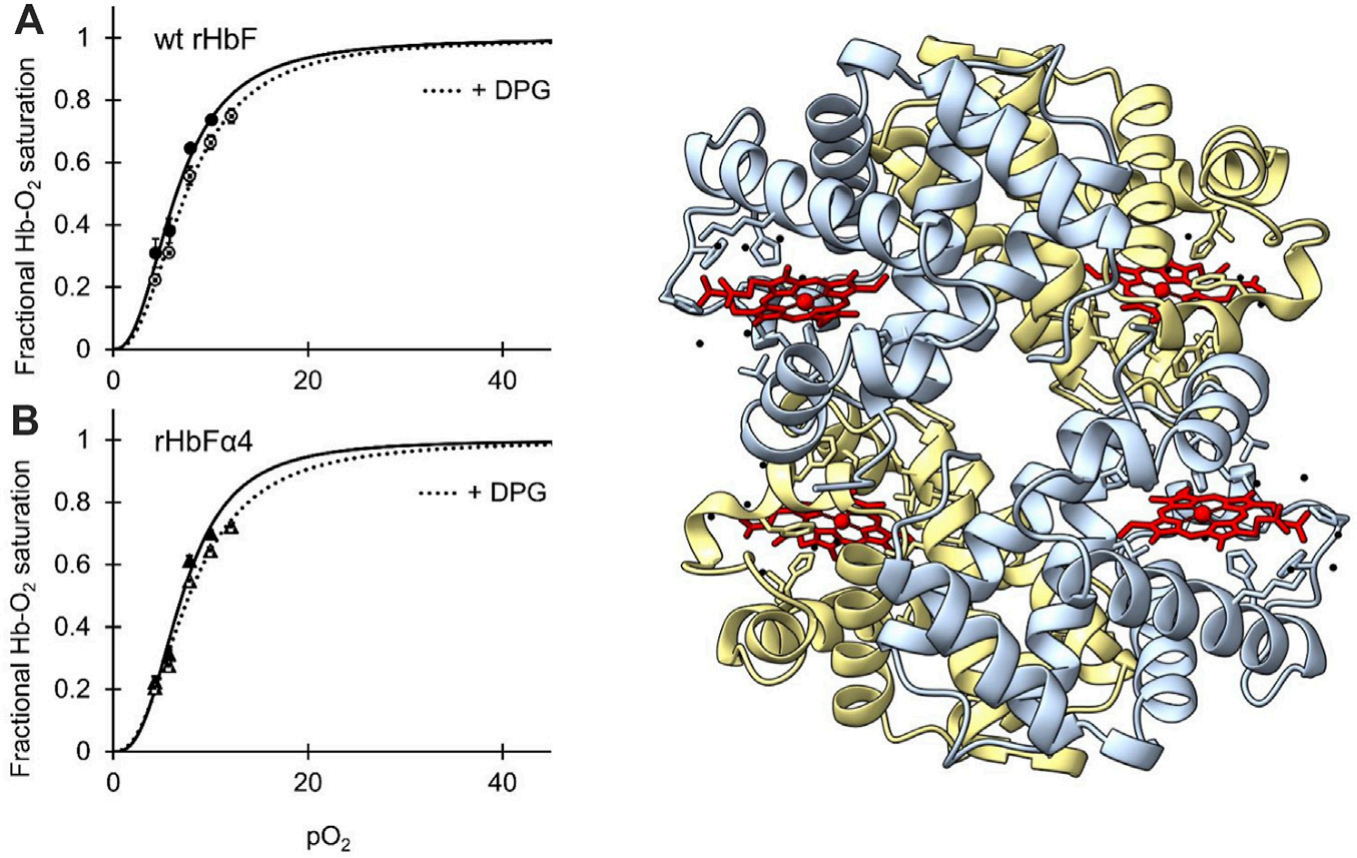
[IN COLOR] Oxygen binding curves of wt HbF **(A)** and HbFα4 mutant **(B)** at 37°C, at a Hb concentration of 0.3 mM heme in 0.1 M HEPES buffer pH 7.4 supplemented with 0.1 M KCl. Solid lines represent the oxygen-binding curve without the addition of DPG, while the dotted lines show the oxygen binding with 0.75 mM DPG present. To the right of the graphs, the 3D structure of mutant rHbFα4 obtained by X-ray crystallography is shown (PDB database entry code: 7QU4). The α-subunits are colored light blue and the γ-subunits are light yellow. The heme groups are shown in red while water molecules are colored black. The figure was created in the UCSF ChimeraX molecular visualization program (Goddard et al., 2018).

**FIGURE 4:**
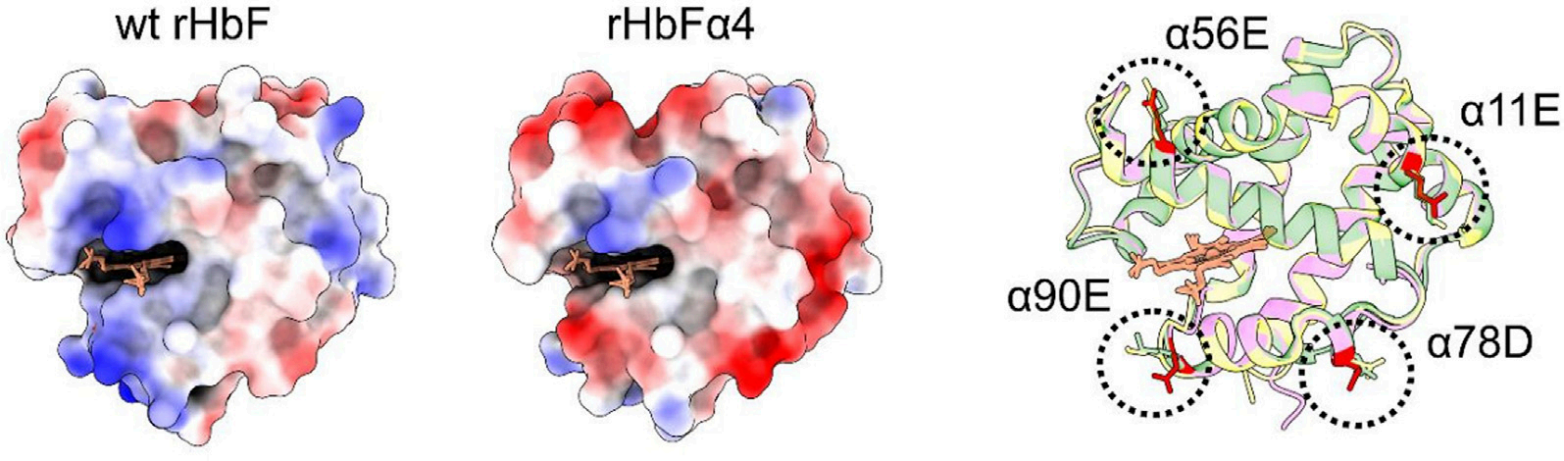
[IN COLOR] The α-subunits of the wt and mutant HbF structures are shown with electrostatic surface coloring. Blue color indicates positive charge and red color indicates negative charge. To the right, three different HbF α-subunit structures, one from this work (rHbFα4 purple) and two from the PDB database (1FDH (Soman and Olson, 2013) yellow, 4MQJ (Miklos et al., 2012) green), are three-dimensionally aligned with the peptide backbones shown as ribbons. The mutation sites of the rHbFα4 mutant are colored in red for clarification. All 3D structures of the α-subunit in HbF align and show that the four mutations introduced on the protein in this work do not disrupt the globin fold.

To examine if the mutations of rHbFα4 affected the 3D structure of HbF we performed X-ray crystallography experiments to determine the protein structure. The overall structure of the mutant HbF crystal was found to be very similar to the wt rHbF and the mutations did not appear to cause any apparent structural perturbations (Figure 3). The statistics and parameters of the crystallographic data processing are presented in Table 3. The 3D structure of this work also compares well to previously reported HbF PDB structures 1FDH (Frier and Perutz, 1977) and 4MQJ (Soman and Olson, 2013). The structure confirms that the mutation sites are located as expected from the initial design idea, and the negatively charged side-chains are positioned on the protein surface as intended. Figure 4 shows the electrostatic surface and the 3D alignment of the α-subunit of the rHbFα4 structure. A closer inspection of the electron densities of each of the residues in the α-subunit revealed that two residues in close proximities to the mutation sites differed between the wt rHbF and the mutant rHbFα4. The charged side-chains of αD75 (EF4) and αH89 (F10) had altered directions in the HbFα4 crystal structure compared to wt HbF. These residues are not in direct contact with heme nor any redox-active residues. This may indicate that any differences seen related to heme or redox rates would be difficult to pinpoint in this type of rigid crystal structure.

**TABLE 3:** X-ray crystallization data collection and refinement statistics of HbFα4 in the Fe^3+^ state.

Crystal data	
Space group	C222_1_
Cell parameters (Å/°)	97.45 189.20 66.61/90.0 90.0 90.0
Wavelength (Å)	0.976
Resolution range (Å)	54.463–1.657 (1.685–1.657)^a^
R_merge_	0.067 (1.337)^a^
No. of observations	989,780 (49,724)^a^
No. of unique observations	72,637 (3,568)^a^
Mean I/σ(I)	21.2 (2.2)^a^
Completeness	98.9 (98.3)^a^
Multiplicity	13.6 (13.9)^a^
CC(1/2)	0.999 (0.774)^a^
Refinement	
Resolution range (Å)	52.946–1.656
R_work_/R_free_	0.1821/0.2194
R_free_ test set, reflections (%)	4.89
No. of non H-atoms	5,098
Ligands	4 heme
*R.m.s deviation from ideal geometry*	
Bonds (Å)	0.008
Angles (°)	0.955
Average B, all atoms (Å^2^)	30.6 (range 16.9–69.1)
Ramachandran plot	
Favored regions (%)	99.29
Allowed regions (%)	0.71
Outliers (%)	0.00
MolProbity clash score	1.56

^a^ Outer resolution shell.

The oxygen binding study showed that the mutations in the rHbFα4 mutant did not affect the oxygen affinity or the cooperativity of the wt HbF protein (Figure 3; Table 4). It was also confirmed that DPG had a little effect on the oxygen binding curves of both the wildtype and the mutant protein, as known for native HbF. This indicated that Hb’s main functionality trait as an oxygen carrier was not compromised by the modified design of the α-subunit.

**TABLE 4:** Oxygen bidning characteristics of native and mutant HbF.

			wt HbF	HbFα4
P50	(*mm Hg*)	+ DPG	6.5 ± 0.3	7.1 ± 0.1
			7.5 ± 0.2	7.8 ± 0.1
n50		+ DPG	2.4 ± 0.3	2.8 ± 0.1
			2.4 ± 0.2	2.4 ± 0.05

## 4 Discussion

Insertion of charged residues has become an attractive strategy for decreasing the aggregation properties of proteins. It may involve single residues or can be more substantial and include so-called “supercharged” proteins to prevent tendencies for aggregation (Miklos et al., 2012). In a heterologous system, the possibilities of using this chaperone are limited and the control of globin chain concentrations can be difficult, if not impossible, to fully achieve. The introduction of negative charges on the less soluble α-subunit promotes the formation of the HbF tetramers and this strategy for increasing HbF expression in *E. coli* represents a simple way to enhance production by at least 2-fold (Kettisen and Bulow, 2021). However, to verify the feasibility of this strategy, the mutated protein must be extensively characterized to ensure that the modifications do not disrupt any critical functions of the protein. In this study, we compared the biochemical properties of a negatively charged and high-yielding HbF mutant with the wt HbF protein. Several characteristics were examined including oxygen binding properties, spontaneous and oxidant/reductant-induced redox reactions, heme release in presence of heme-scavenging myoglobin, DNA cleavage activity, thermal denaturation, and plasma clearance in mice in a top-load experiment. To verify that no structural perturbations occurred upon including more negative charges on the protein surface, we also examined the ability to bind to the Hb-scavenger Hp, as well as determined the 3D structure of the mutant HbF. The long-term aim is to use this HbF mutant for oxygen therapeutics applications by entrapping the protein in lipid vesicles (Buehler et al., 2010).

### 4.1 Redox reactions reveal differences in rates of reduction by the mutant HbF with ascorbate

The reaction rates towards higher oxidation states of the heme iron (Fe^3+^ and Fe^4+^) did not differ between the wt rHbF and the mutant, but reducing the heme iron back to lower oxidation states with a reducing agent revealed that the mutant reacted in a slightly different way. Ferryl reduction is governed by different kinetic phases–a high and a low-affinity pathway, related to the different pathways of electron transfer, through-protein electron “hopping” and direct heme reduction, respectively (Reeder et al., 2008). Within the range of ascorbate concentrations applied in this study, we found that the kinetic phase governed by the α-subunit appeared to be less effective in utilizing the high-affinity pathway in the mutant, as revealed by the decreased k_max_/K_D_ value compared to wt rHbF. As this pathway is assumed to be dependent on tyrosine residues in the vicinity of the heme group to act as an electron conduit, this could be due to some structural change affecting the αY42 residue position originating from the selected mutations. Another difference seen in the ferryl reduction experiment related to the α-subunit was the increased low-affinity pathway reduction rate, which was increased 3-fold. As this pathway is believed to be governed by the direct reduction of the heme by the reductant, it could mean that there is increased access to the heme in the mutant protein. A further indication of possible rearrangement of residues able to affect the heme-related reactions was that the autoreduction of the ferryl state to the ferric state was increased in the mutant. Coupled with the heme loss study indicating a slightly increased heme loss from the α-subunit, small structural changes in the heme pocket vicinity may be considered to be responsible for the observed differences. However, the X-ray crystallographic analysis showed that the 3D structures of wt rHbF and rHbFα4 were almost identical and no apparent changes in the structural configuration of the peptide backbones could be observed. The difference in charge distribution coming from the mutation sites α78 and α90 may have had an impact on the charged residues of αD75 in the EF corner and αH89 at the end of helix F, as the side-chains of these residues appeared to be able to align differently in the rHbFα4 crystal structure. The modification of charge distribution on the Hb surface could very well affect the heme surroundings in a subtle way, which unfortunately appears to be hard to pinpoint from the crystal structure. The environment in which protein crystals are formed could be quite different from the native conditions of a protein, which might affect the exact configuration of the crystallized protein, and especially the more flexible parts. Evidently, from the differences seen in the redox studies in this work, the rHbFα4 appears to have some minor alterations that influence the reactions rates when the heme iron is reduced from a higher oxidation state compared to wt rHbF.

### 4.2 DNA cleavage activity was greatly reduced with the mutant HbF compared to wild type

Important biomolecules, such as nucleic acids, are often subjected to hydrolytic and oxidative side reactions (Simunkova et al., 2019). The oxidative activities of Hb have been shown to readily cleave supercoiled plasmid DNA (Chakane et al., 2017). In a previous study, we reported that the addition of 1-2 negative charges successfully decreased the DNA cleavage rate of HbF (Kettisen et al., 2021). Here, the cleavage rates of supercoiled plasmid DNA at increasing concentrations of Hb were analyzed and compared. The rate constant was four times faster with the wt rHbF compared to the mutant, which showed that the negatively charged surface-exposed residues on the mutant decidedly contributed to a reduced rate of degradation of the negatively charged backbone of plasmid DNA. This can be an advantage during the expression of the mutant compared to wt rHbF and could be an additional contributing factor to the increased yield seen in *E. coli* with this mutant. The expression plasmids coding for Hb in heterologous hosts may be affected by increasing concentrations of Hb, especially in high-yield fermentation, and decreasing the effect of such harmful reactions should be attractive to ensure efficient production levels. This study shows that the strategy of adding to the negative net charge on the surface of HbF considerably contributed to a slower rate of Hb-induced DNA damage.

### 4.3 Oxygen binding, thermal denaturation, haptoglobin binding, and plasma half-life are not affected by the mutations in the HbF mutant

Mutations in a multimeric and cooperative protein like Hb may cause an unintended impact on the protein functionality. Four mutations on the α-subunit result in eight positions in total on the complete assembled tetramer and will lead to a substantially changed electrostatic nature. Thermal denaturation profiles of the proteins were determined in a concentrated solution of Hb (188 µM heme) to ensure stable Hb tetramers. We could not observe any significant differences between the wt and the mutant in this work. However, the mutant showed much clearer transitions than wt rHbF. As the instrument measures the general signal of fluorescent residue side-chains, the increased ratio amplitude changes in the mutant HbF might indicate a difference in exposure of these side chains. However, as discussed previously, the crystal structures solved in this work showed no indication of apparent structural differences, also regarding the aromatic side-chains of Phe, Tyr, and Trp in the mutant. Considering the locations of the mutations, the αD78 residue is close to αY140 and could perhaps influence the location of the C-terminal residues in solution, and the αE56 residue on the E-helix might be able to affect αE23 on the B-helix which is close to αY24. Nevertheless, even though the amplitude of the fluorescence ratio changes might suggest some difference in aromatic side-chain exposure between the HbF variants, the onset and transition temperatures were similar between wt rHbF and the mutant, and thus indicated no difference in overall thermal stability.

In the size-exclusion experiments with or without Hp, it was found that Hp efficiently could capture both the wt rHbF and the mutant. The mutations in the rHbFα4 thus did not affect the dimer interface responsible for Hp binding (Alayash et al., 2013; Ratanasopa et al., 2013). Similarly, the plasma half-life of the mutant HbF was comparable to previously reported values in mouse models (Kettisen et al., 2021; Bobofchak et al., 2003). Without other retention time-extending engineering strategies, for example, cross-linking, polymerization, or genetic fusion of subunits/functional polypeptide tags, the Hb will be removed rapidly from circulation. We previously observed a slightly extended half-life with another mutant of HbF where non-polar alanine residues were substituted for aspartate on the α-subunit (Kettisen et al., 2021). However, the mutant in this work where mainly positive charges were substituted into negative charges, the total number of surface charges were not changed. Although the increased negative charge of the rHbFα4 mutant in this work was hypothesized to possibly influence the interaction with the negatively charged fine mesh that makes up the selective filtration in the glomerular filtration barrier (Bunn et al., 1969; Schlöndorff et al., 2017), it is concluded that this strategy cannot be properly evaluated unless combined with a protein design where the tetrameric form of Hb is ensured. The strategy of switching or adding surface charges may thus still be worthwhile to explore further. The obtained results are promising, but to fully elucidate the influence of charged Hb proteins on the expression in heterologous host systems, redox properties, and *in vivo* physiological behaviors, more studies deserve to be carried out.

## Data Availability

The original contributions presented in the study are included in the article/Supplementary Material, further inquiries can be directed to the corresponding authors.
