# Supplementary material for: Structural and oxidative investigation of a recombinant high-yielding fetal hemoglobin mutant
Source: Front Mol Biosci. 2023 Mar 16;10:1133985. doi: 10.3389/fmolb.2023.1133985 (PMC10060959; doi:10.3389/fmolb.2023.1133985)
Supplement: Supplementary file 1 [file Table1.docx]

**Supplementary Material**

Structural and Oxidative Investigation of a Recombinant High-Yielding Fetal Hemoglobin Mutant

## 1 Supplementary data


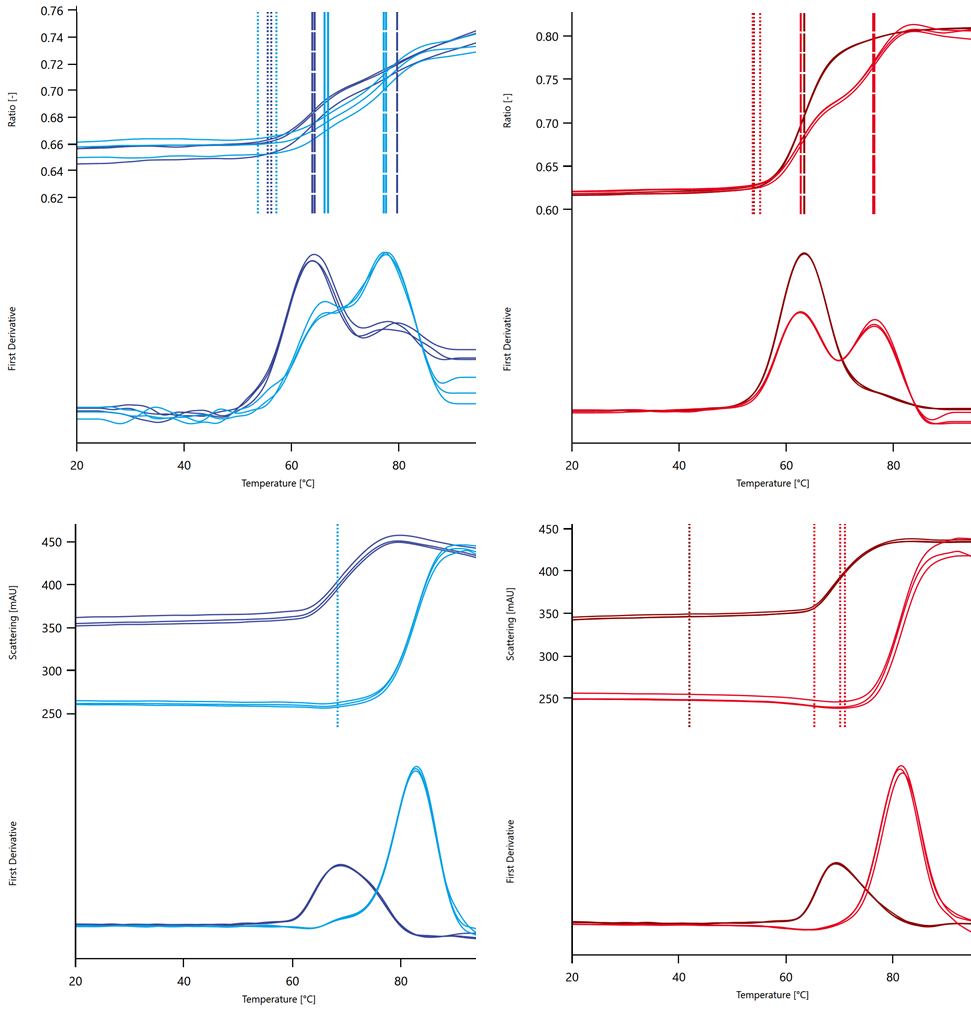


**Figure S1**. Thermal denaturation of HbF as measured by DSF in a Prometheus NT.48 instrument (NanoTemper Technologies). Hb samples (blue – wt rHbF, red – mutant) in 100 mM phosphate buffer pH 7.4, either bound to O_2_-bound (darker lines) or CO (lighter lines) were subjected to a temperature ramp of 7 °C/min. The upper graphs show the ratio 350/330 nm and the lower graphs the corresponding scattering curves.


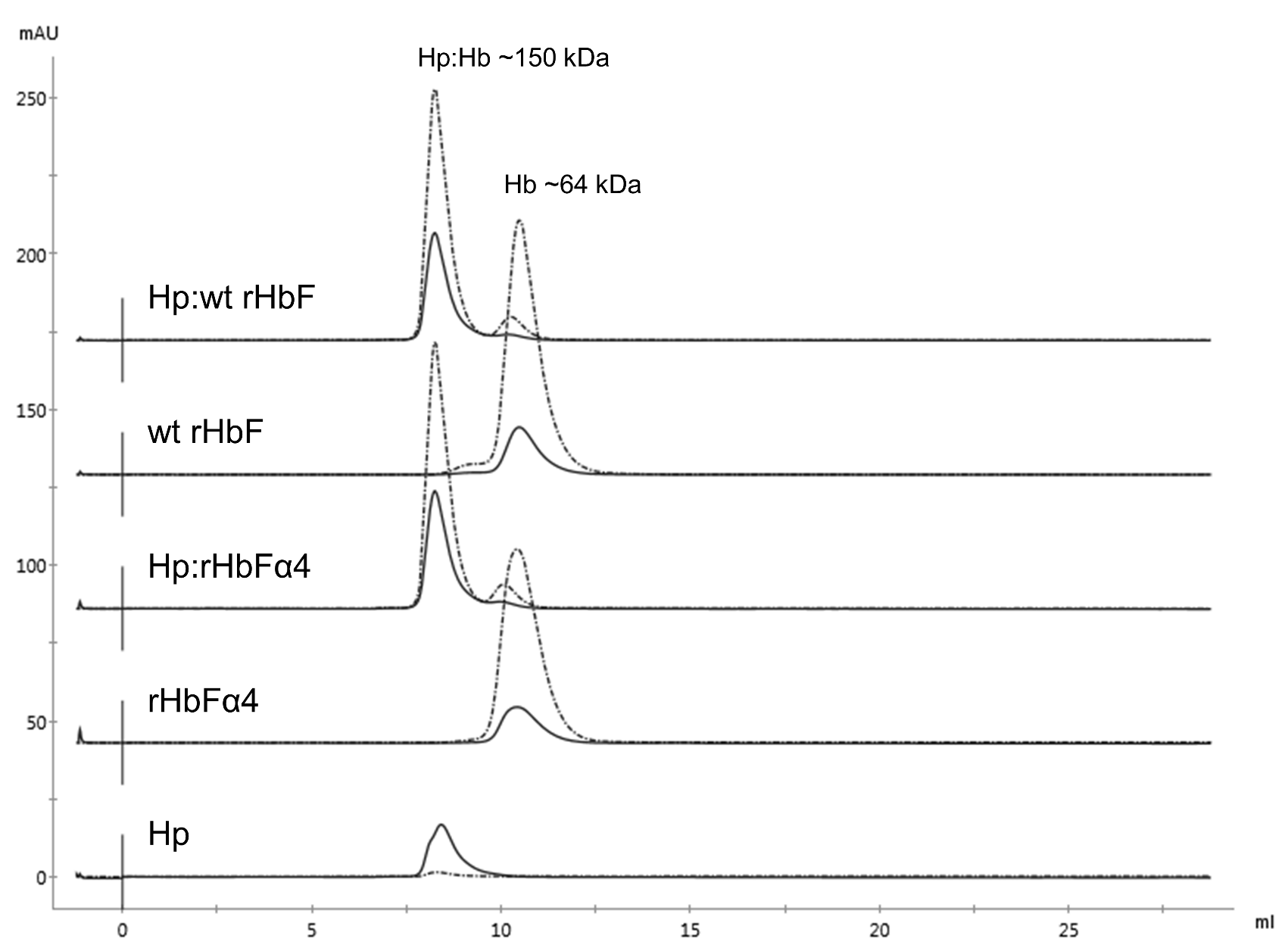


**Figure S2**. Binding of HbF to Hp. Chromatograms of wt rHbF and the rHbFα4 mutant applied to a Superdex^®^ 75 10/300 GL column (GE Healthcare) in 50 mM sodium phosphate buffer pH 7.2, supplemented with 150 mM NaCl. Solid lines show the 280 nm and the dashed lines show the 419 nm UV trace. Ferric Hb was mixed with Hp in a 1:1 molar ratio and incubated at 37 °C for 30 min before injection on the column. The chromatographic separation was run at a flow rate of 0.5 ml/min.


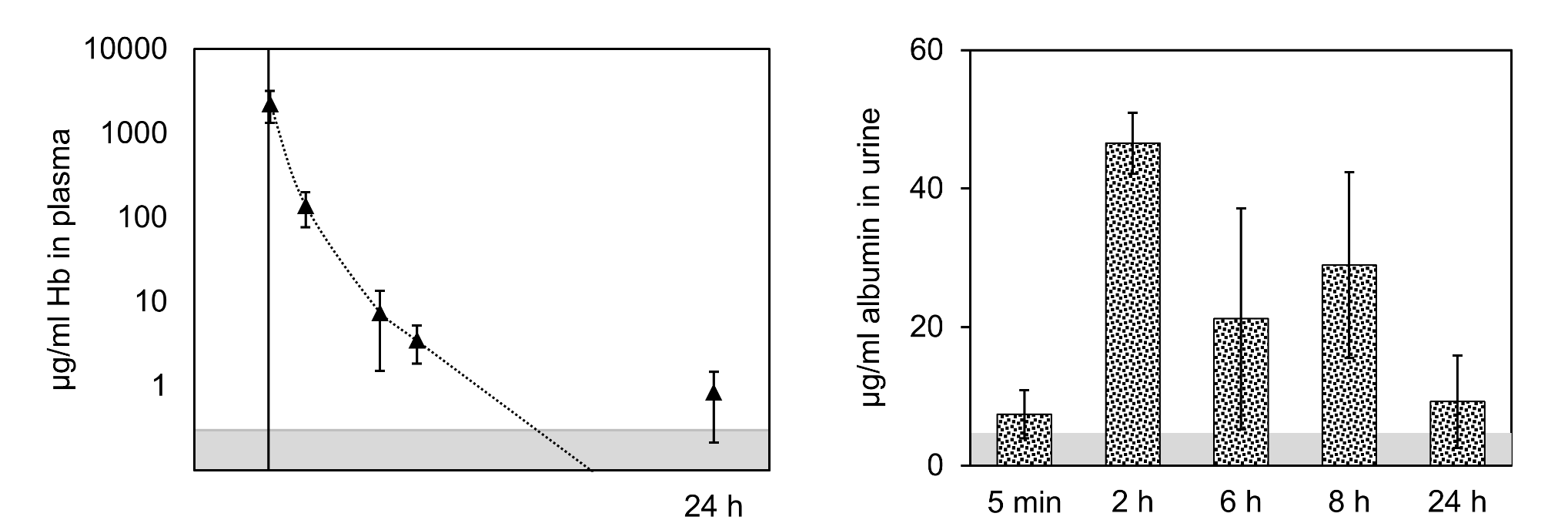


**Figure S3.** The pharmacokinetics of the rHbFα4 mutant was analyzed by sandwich ELISA in plasma samples at five different time-points (5 min, 2 h, 6 h, 8 h, and 24 h, n = 5 per time point). The albumin assay of the urine samples (n = 3-5) showed that the concentration of albumin was increased in the urine at the 2 h, 6 h, and 8 h time points, but not significantly different from non-injected control levels at 24 h. The data is presented as mean ± SD. Differences are determined by independent t-tests with p < 0.05 significance level. The grey area shown in the graphs indicates the signal of the non-injected control (n = 5).
